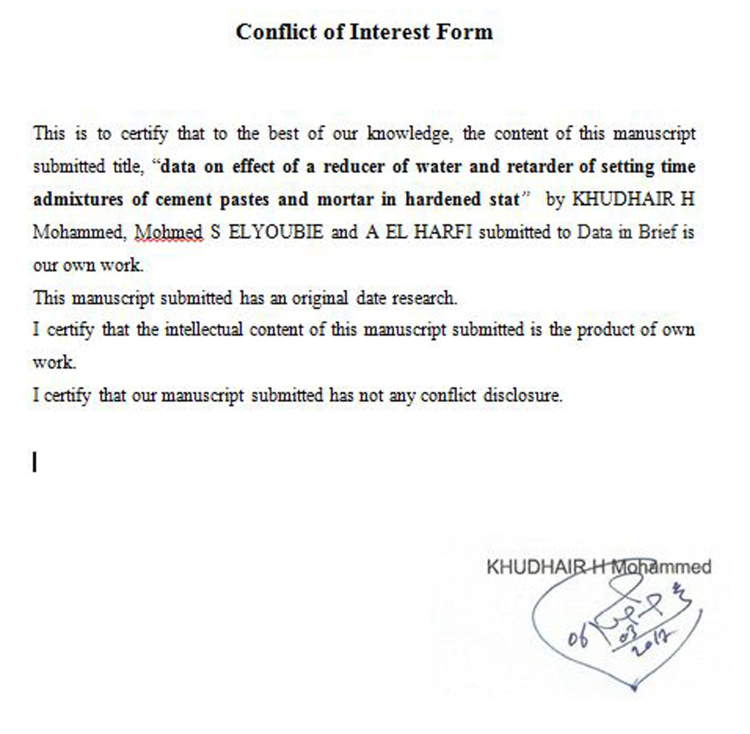# Data on effect of a reducer of water and retarder of setting time admixtures of cement pastes and mortar in hardened stat

**DOI:** 10.1016/j.dib.2018.03.050

**Published:** 2018-03-17

**Authors:** Mohammed Husssein Khudhair, Mohamed S. El Youbi, Ahmed Elharfi

**Affiliations:** a Laboratory of Agro resources Polymers and Process engineering (LAPPE), Team of Macromolecular & Organic Chemistry, Faculty of Sciences, Ibn Tofail University, BP 133, 14000 Kenitra, Morocco; b Laboratory of Cement and Quality Control of Amran Cement Plant of Yemen, Yemen; c Laboratory of Chemistry of Solid State, Faculty of Science, Ibn Tofail University, Kenitra, Morocco

**Keywords:** Superplasticizers, Reducer of water, Durable concrete, Retarder of setting, Physical properties, High performance, Compressive strength

## Abstract

The aim of this work is to study the influence of the admixture of superplasticizers on the physical properties of cement paste to know the initial and final time, water content and mechanical performance of concrete.

In this work we have incorporated an Advanced Superplasticité for Prolonged Slump Retention (ASPPSR402) in the formulation matrix of concrete at different percentages ranging from 0.5% to 5% by weight of cement with a step of 0.5%, while partially substituting the mixing water by the last one to reduce the amount of water used.

The obtained results by different prospected formulations show that the admixture of ASPPSR402 in formulations matrix significantly reduces the (Water/Cement “W/C”) ratio. Subsequently, we observed that the initial and final time increases in function of the percentage of ASPPSR402. Similarly, the compressive strength at the young age, median age and long-term has been improved.

**Specifications Table**

**Table:** 

*Subject area*	*Civil Engineering, Material Science Engineering*
*More specific subject area*	*Portland cement, Cement based admixture concrete,*
*Type of data*	*Table, image, text file, graph and figure*
*How data was acquired*	*Physical and mechanical tests (Laboratory): X-Ray Fluorescence (XRF), grading of sand, fresh cement paste, WC ration, Setting time, compressive strength*
*Data format*	*Raw and analyzed data*
*Experimental factors*	*The ten different volume fractions of advanced admixtures of superplasticizer for prolonged slump retention and cement used to manufacture the cement paste composites and mortar or concrete in a small mold.*
*Experimental features*	*Various volume of advanced admixtures of superplasticizer for prolonged slump retention are blended with Portland cement and water in the presence of standard sand for prepare a paste composites and mortar or concrete to investigate theme physical and mechanical properties*
*Data source location*	*Laboratory of cement and quality control of Amran cement plant (Yemen) in collaboration with the laboratory of agro-resources polymers and process engineering of the faculty of science, Ibn Tofail University (Kenitra-Morocco)*
*Data accessibility*	*Data provided in the article is accessible to the public*

**Value of the data**

● Evaluation of the W/C ration, setting time and the compressive strength of Portland cement (pastes cement, mortar or concrete) compared to the cement (pastes cement, mortar or concrete) based on the various volume of advanced admixtures of superplasticizer for prolonged slump retention.
● Estimation of the evolution of the ratio (W/C) and the initial and final time of cement paste as a function of the various percentages of the ASPPSR402 and the amount of water reduced according to the various percentages of ASPPSR402.
● Estimation the rate of change compressive strength of ordinary Portland cement as a function of curing time.
● Optimization of the curing time to achieve a particular rate of change of compressive strength of using the admixture of ASPPSR402.

## 1 Data

The data of the different formulations in a fresh and hardened state presented here are from eleven different cement pastes, mortar or concrete samples fabricated to compare cement pastes, mortar or concrete based of ASPPSR402 with Portland cement based concrete.

## 2 Sample preparation method

To achieve the objective of our study, we made a mortar of reference without additions and ten samples with the ASPPSR402 whose compositions are inspired by that of the normal mortar defined by EN196-1, with a quantity of water adjusted to obtain a paste with a standard consistency. The procedures followed for the preparation of the pastes cement has been done in maintaining fixed consistency standardized of all the formulations produced according to the specification of the standard (EN 196-3 + A1) [1] and in modifying the ratio W/C. This experiment has achieved using the Vicat apparatus according to the standard (EN 197-1) [2] on one hand and on the other hand, the initial and final time of the cement paste has been studied using the Vicat apparatus according to the specification of the European standard EN 196-3.

To complete our work, we also studied the influence of the ASPPSR402 on the mechanical performance of mortar or concrete by using the compressive strength. The measures of the compressive strength are on standard prismatic standard (4 × 4 × 16) cm^3^ according to the specification of the standard (NF EN 196-1) [3] at different percentages of ASPPSR402, ranging from 0.5% to 5% by weight of cement with a step of 0.5%. These prismatic standards are removed after one day and kept under water for the period of crushing. The measure of the compressive strength was performed at young age "2 days", "7 days" medium-term and long-term "28 days" in order to observe the gradual evolution of the mechanical performance of our formulations using ASPPSR402, based on time in days.

The compositions of the different formulations in a fresh and hardened state are recorded in the (Table 1, Table 2).

**Table 1:** Matrices of formulations of fresh cement paste with the ASPPSR402.

**Order number of samples**	**Mass of cement (g)**	**Water (ml)**	**W/C**	**ASPPSR402%**
1	500	130	0.26	
2	500	120	0.24	0.50
3	500	115	0.23	1.00
4	500	110	0.22	1.50
5	500	105	0.21	2.00
6	500	100	0.2	2.50
7	500	95	0.19	3.00
8	500	90	0.18	3.50
9	500	84	0.168	4.00
10	500	84	0.168	4.50
11	500	84	0.168	5.00

**Table 2:** Matrices of formulations of the mortar of cement with ASPPSR402 in the hardened state.

**Order number of samples**	**Mass of cement (g)**	**Water (ml)**	**ASPPSR402%**	**Sand (g)**	**W/C**
1	450	225	0.00	1350	0.50
2	450	211,5	0.50	1350	0.47
3	450	200	1.00	1350	0.44
4	450	188	1.50	1350	0.42
5	450	174	2.00	1350	0.39
6	450	160	2.50	1350	0.36
7	450	155	3.00	1350	0.34
8	450	144	3.50	1350	0.32
9	450	141	4.00	1350	0.31
10	450	140	4.50	1350	0.31
11	450	140	5.00	1350	0.31

## 3 Characterization and data analysis

### 3.1 Characterization and data analysis of materials used

For evaluating the influence the Admixtures of advanced superplasticiser for prolonged slump retention "ASPPSR402", on physical properties of fresh cement paste and mechanical performance (mechanical resistance to compression) of mortars and /or concretes in the hardened State. We have preceded our work by the characterizing of materials used, to understand the phenomena which occur at the moment of mixing and hardening of concrete.

#### 3.1.1 Cement

The type of cement used in this work is (CMI/42.5) from the plant of Amran in Yemen. The chemical and mineralogical compositions of clinker, gypsum and cement determined by XRF are presented in the Table 3, Table 4:

**Table 3:** Elementary chemical compositions of clinker, gypsum and cement.

**Chemical name**	**Chemical formula**	**Cement nomenclature**	**Clinker**	**Gypsum**	**Cement**
**Lime**	CaO	C	62.76	33.40	61.29
**Silica**	SiO_2_	S	21.00	0.70	19.99
**Alumina**	Al_2_O_3_	A	5.84	0.36	5.57
**Ferrite**	Fe_2_O_3_	F	3.00	0.09	2.85
**Magnesia**	MgO	M	1.96	0.63	1.89
**Sulfur trioxide**	SO_3_	Ś	0.90	47.20	3.22
**Potassium oxide**	K_2_O	K	1.21	0.03	1.15
**Sodium oxide**	Na_2_O	N	0.20	0.10	0.20
**Chloride ion**	Cl^−^	Cl	0.02	0.01	0.02

**Table 4:** Mineralogical composition of clinker.

**Chemical name**	**Mineral name**	**Chemical formula**	**Cement nomenclature**	**Content**
**Tricalcium silicate**	Alite	Ca_3_SiO_5_	C_3_S	47.70
**Dicalcium silicate**	Balite	Ca_2_SiO_4_	C_2_S	25.10
**Aluminate tricalcium**	Aluminate	Ca_3_Al_2_O_6_	C_3_A	10.40
**Tetracalcium Aluminoferrite**	Ferrite	Ca_4_Al_2_Fe_2_O_10_	C_4_AF	9.10

##### 3.1.1.1 Physical properties

The physical properties of clinker and cement are gathered at the Table 5:

**Table 5:** Physical properties of clinker.

**Designations**	**Values**		**Units**
	**Clinker**	**Cement**	
Absolute density	3.16	3.14	g cm^−3^
Refusal of the sieve 45 μm	11.60	12.50	%
Refusal of the sieve 90 μm	1.24	1.50	%
Specific surface Blaine	3360.00	3240.00	cm^2^ g^−1^

#### 3.1.2 Sand

**Fig. 1:**
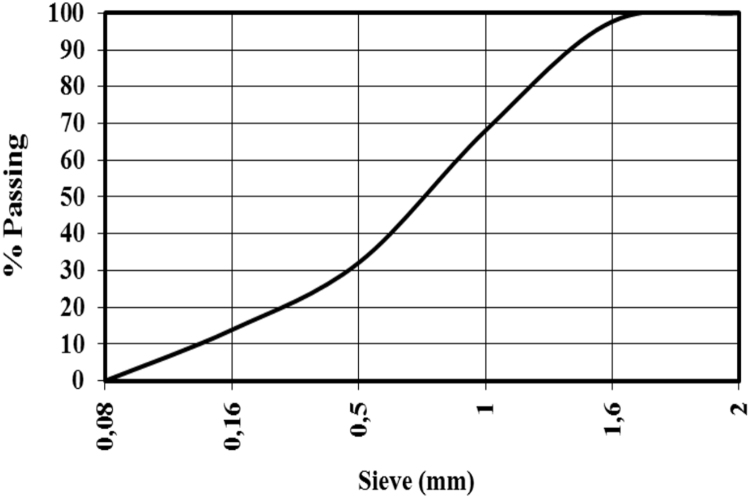
Grading curve of sand.

To prepare our mortar, we used a standard sand conferring to the standard EN 196-1 [4], delivered by the new French company of the Littoral. The analysis of the particle size of sands is illustrated in the Fig. 1.

#### 3.1.3 Adjuvant (superplasticizer)

**Fig. 2:**
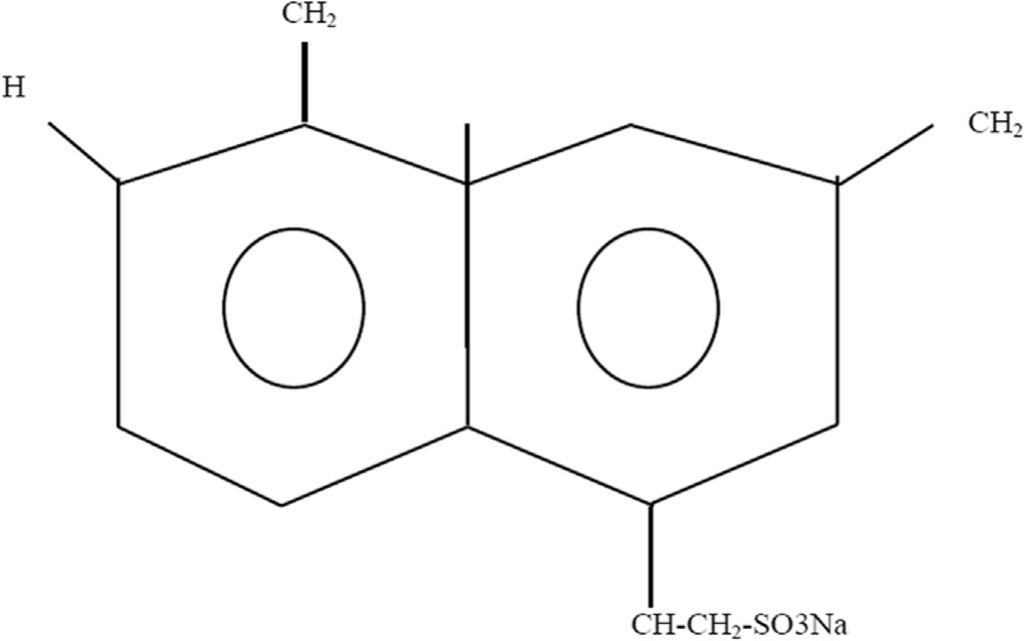
Poly-naphthalene sulfone.

**Fig. 3:**
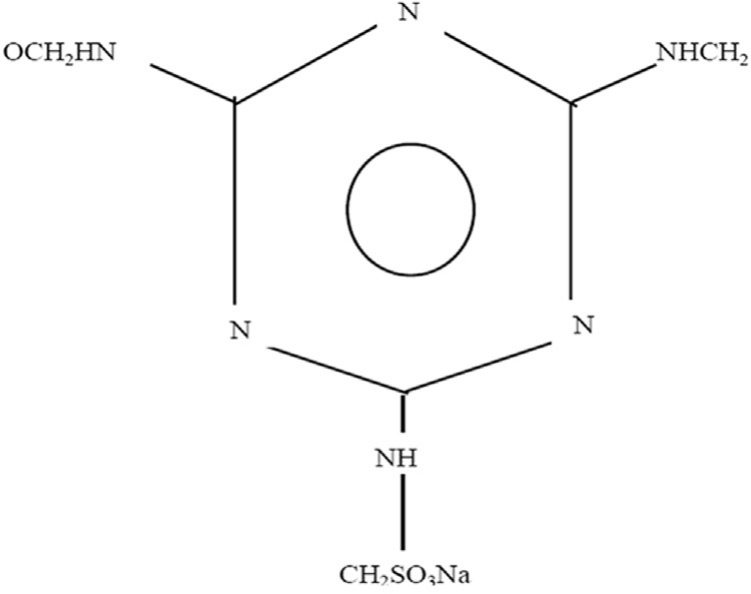
Poly melamine sulfone.

**Fig. 4:**
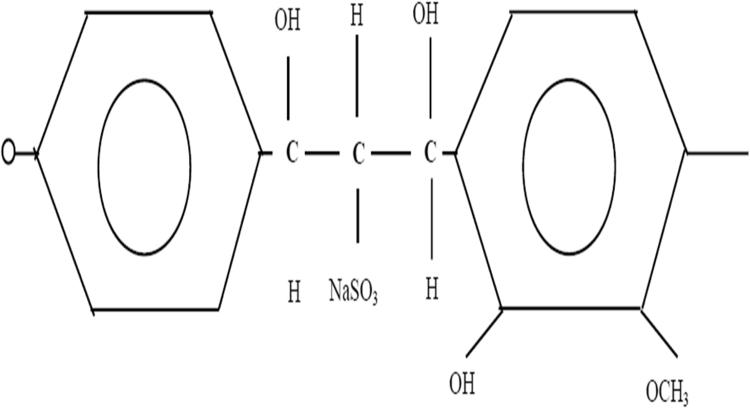
Lignosulphonate of high purity.

The Advanced superplasticizer for Prolonged Slump Retention "ASPPSR402"), are polymers in liquid form, prepared especially for the cement industry and concrete. They are a basis of salts of sodium or calcium of poly-naphthalene sulfone (Fig. 2), salt of sodium of poly melamine sulfone (Fig. 3), acrylate-ester (polycrylate) copolymer, or lignosulphonate of high purity (Fig. 4) [5], [6].

The ASPPSR402 used in the formulation of mortars and/or concrete of high performance, delivered by the company CONMIX Ltd in Sharjah, United Arab Emirates. They are incorporated during the mixing of the concrete in equal a dose raining from 0.5% to 5% by weight of cement with a step of 0.5% to improve the physical and the mechanical properties of fresh or hardened State.

The physical properties of ASPPSR402 are collected at the Table 6:

**Table 6:** Physical properties of superplasticizer ASPPSR402.

**Name**	**Nature**	**Color**	**Density (g cm**^**−3**^**)**	**Area training (%)**	**Chloride content**
**ASPPSR402**	Liquid	Brown	1.23	0.5–1.0	Nile

#### 3.1.4 The mixing water

To waste our mixture, we used tap water (wells). The physical properties of mixing water are assembled at the Table 7:

**Table 7:** Main features of the mixing water.

**Components**	**Units**	**Values**
**pH**	–	7.00
**Turbidity**	(mg/l)	450.00
**CO**_**3**_^**−2**^	(mg/l)	216.00
**HCO**_**3**_^**−**^	(mg/l)	0.00
**Ca**^**+2**^	(mg/l)	56.40
**Mg**^**+2**^	(mg/l)	52.40
**Conductivity**	µS/cm	692.00

### 3.2 Characterization and data analysis of pastes cement

#### 3.2.1 Data analysis of the influence of the ASPPSR402 on W/C ratio

**Fig. 5:**
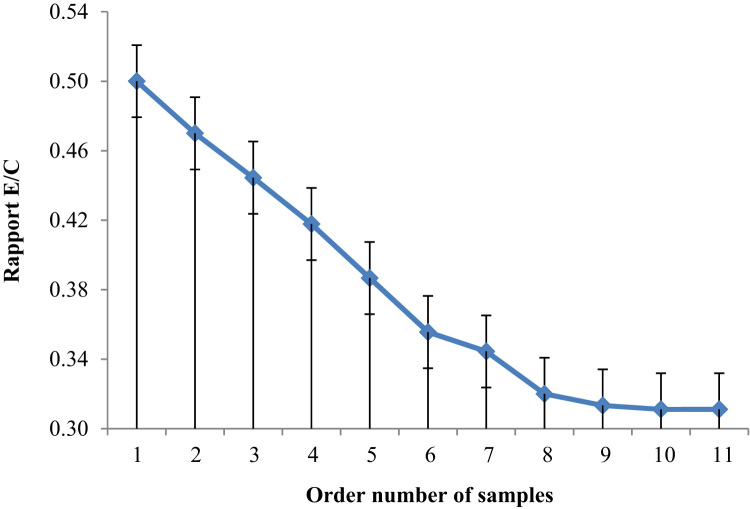
Variation of W/C ratio of cement as a function of order number of samples.

**Fig. 6:**
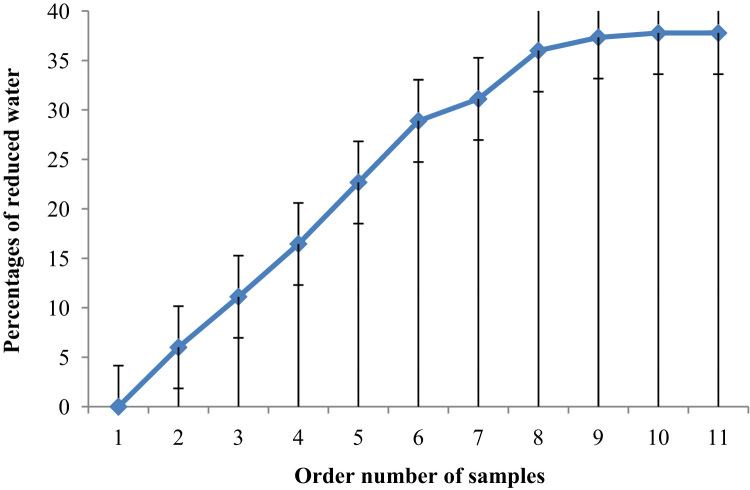
The amount of water reduced as a function of order number of samples.

The Fig. 5, Fig. 6, show the W/C ratio and always the quantity of water reduced as a function of the dosage of ASPPSR402.

#### 3.2.2 Data analysis of the influence of the ASPPSR402 on setting time

**Fig. 7:**
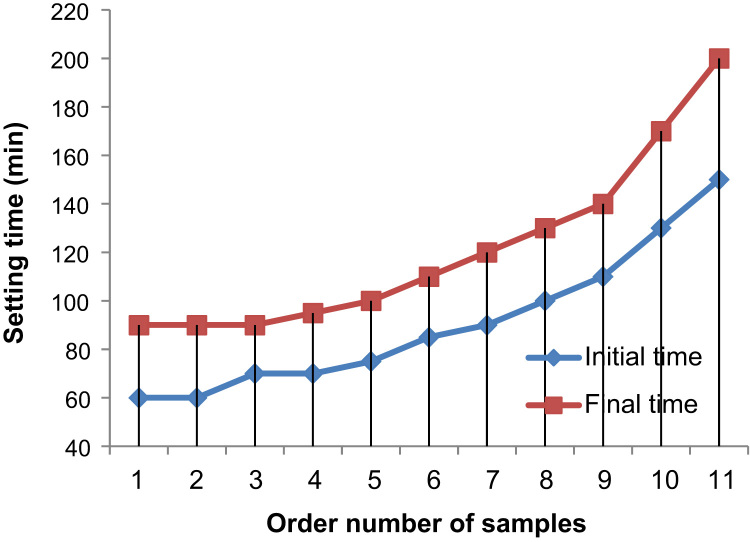
Evaluation of setting time as a function of the percentage of ASPPSR402.

The Fig. 7 reveals the evolution of the time as a function of the different percentages of ASPPSR402.

### 3.3 Data analysis of the influence of the ASPPSR402 on hardened state of mortar of concrete

**Fig. 8:**
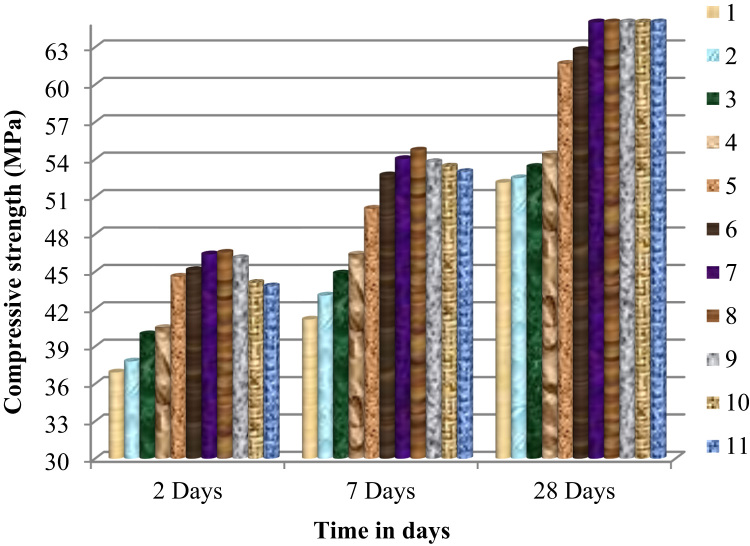
Variation of the compressive strength of mortar or concrete as a function of time in days.

The Fig. 8 presents the evolution of the compressive strength as a function of time in days.

**Fig. 9:**
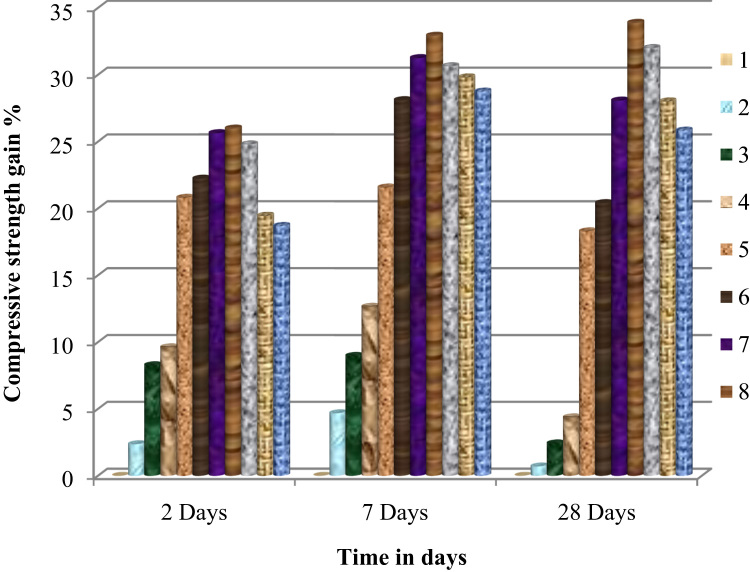
Variation of gain in compressive strength of mortar or concrete as a function of time in days.

The Fig. 9 presents the gain in ' R_MC_' at different percentages of ASPPSR402.

## Figures and Tables

**Fig. S1:**